# Self-sufficient primary natural killer cells engineered to express T cell receptors and interleukin-15 exhibit improved effector function and persistence

**DOI:** 10.3389/fimmu.2024.1368290

**Published:** 2024-04-16

**Authors:** Els P. van Hees, Laura T. Morton, Dennis F. G. Remst, Anne K. Wouters, Astrid Van den Eynde, J. H. Frederik Falkenburg, Mirjam H.M. Heemskerk

**Affiliations:** ^1^ Department of Hematology, Leiden University Medical Centre (LUMC), Leiden, Netherlands; ^2^ Center for Oncological Research (CORE), Integrated Personalized and Precision Oncology Network (IPPON), Antwerp, Belgium

**Keywords:** immunotherapy, NK cells, T-cell receptor, cancer, multiple myeloma, Bob1, interleukin-15

## Abstract

**Background:**

NK cells can be genetically engineered to express a transgenic T-cell receptor (TCR). This approach offers an alternative strategy to target heterogenous tumors, as NK:TCR cells can eradicate both tumor cells with high expression of HLA class I and antigen of interest or HLA class I negative tumors. Expansion and survival of NK cells relies on the presence of IL-15. Therefore, autonomous production of IL-15 by NK:TCR cells might improve functional persistence of NK cells. Here we present an optimized NK:TCR product harnessed with a construct encoding for soluble IL-15 (NK:TCR/IL-15), to support their proliferation, persistence and cytotoxic capabilities.

**Methods:**

Expression of tumor-specific TCRs in peripheral blood derived NK-cells was achieved following retroviral transduction. NK:TCR/IL-15 cells were compared with NK:TCR cells for autonomous cytokine production, proliferation and survival. NK:BOB1-TCR/IL-15 cells, expressing a HLA-B*07:02-restricted TCR against BOB1, a B-cell lineage specific transcription factor highly expressed in all B-cell malignancies, were compared with control NK:BOB1-TCR and NK:CMV-TCR/IL-15 cells for effector function against TCR antigen positive malignant B-cell lines *in vitro* and *in vivo.*

**Results:**

Viral incorporation of the interleukin-15 gene into engineered NK:TCR cells was feasible and high expression of the TCR was maintained, resulting in pure NK:TCR/IL-15 cell products generated from peripheral blood of multiple donors. Self-sufficient secretion of IL-15 by NK:TCR cells enables engineered NK cells to proliferate *in vitro* without addition of extra cytokines. NK:TCR/IL-15 demonstrated a marked enhancement of TCR-mediated cytotoxicity as well as enhanced NK-mediated cytotoxicity resulting in improved persistence and performance of NK:BOB1-TCR/IL-15 cells in an orthotopic multiple myeloma mouse model. However, in contrast to prolonged anti-tumor reactivity by NK:BOB1-TCR/IL-15, we observed in one of the experiments an accumulation of NK:BOB1-TCR/IL-15 cells in several organs of treated mice, leading to unexpected death 30 days post-NK infusion.

**Conclusion:**

This study showed that NK:TCR/IL-15 cells secrete low levels of IL-15 and can proliferate in an environment lacking cytokines. Repeated *in vitro* and *in vivo* experiments confirmed the effectiveness and target specificity of our product, in which addition of IL-15 supports TCR- and NK-mediated cytotoxicity.

## Introduction

Over the last decade, several cell-based immunotherapeutic products using genetically engineered T cells with a chimeric antigen receptor (CAR) have been approved by the FDA (1). However, despite their anti-tumor activity, these T cell therapies also have their limitations. First, CAR T cell therapy is associated with an increased risk of toxicity, including cytokine release syndrome (CRS) and neurotoxicity (2). Secondly, the need for individualized T cell engineering is a significant drawback since endogenous T cell receptor (TCR) expression in donor-derived off-the-shelf T cells can lead to graft-versus-host disease (GvHD). This demands the engineering of a unique T cell product that is HLA-matched for each individual patient.

To overcome these limitations, the use of donor-derived natural killer (NK) cells for adoptive transfer might be a potential solution due to their unique cellular properties. The first-in-human clinical trial of a partially HLA-matched NK:CAR cell therapy demonstrated no adverse events such as CRS, neurotoxicity, or GvHD (3). In addition, trials with haploidentical NK cells did not induce GvHD, positioning NK cells as a promising off-the-shelf cellular therapeutic (4).

Recently we have demonstrated that primary NK cells can be genetically engineered to express a transgenic TCR (NK:TCR) (5). This approach offers a strategy to target heterogenous tumors alternative to NK:CAR products. Whereas NK:CAR products target proteins on the cell surface, NK:TCR cells recognize peptides derived from intracellular and extracellular proteins presented in surface expressed HLA, broadening their range of potential targets (6, 7). Therefore, introducing the TCR allows NK:TCR cells to eradicate both tumor cells with high expression of HLA class I and the target antigen, as well as HLA class I-negative tumors (5, 8, 9). This dual-targeting mechanism circumvents a common immune evasion strategy seen in tumors targeted by T cells.

A potential limitation of NK:TCR therapy could be the short lifespan of NK cells resulting from their dependence on interleukin-15 (IL-15) for differentiation, proliferation and persistence (10). However, IL-15 is found at a low basal level in normal human circulation and tissue, therefore autonomous production of IL-15 by NK:TCR cells may be required to persuade functional persistence of NK cells. In addition, the local production of soluble IL-15 in the tumor micro environment (TME) may be a critical component to increase antitumor immunity by activating the already present tumor reactive lymphocytes (11, 12).

So far, clinical trials with NK : CAR cells producing IL-15 have not shown adverse events related to autonomous cytokine production (3, 13). Nevertheless, safety concerns about autonomous cytokine production remain, based on pre-clinical studies with IL-15 engineered T and NK cells, which reported clonal expansion, adverse events and preliminary deaths in mouse models (14–17).

In this study we describe a two-step retroviral transduction protocol for functional TCR expression in primary NK cells that autonomously produce IL-15, resulting in superior performance of engineered NK:TCR/IL-15 cells. However, uncontrolled NK cell proliferation observed in one of the *in vivo* experiments highlights the importance of careful consideration and potentially more quality control for cytokine engineered lymphocytes before moving to clinical application.

## Materials and methods

### NK cell isolation and modification

Peripheral blood was obtained from healthy donors after informed consent. Peripheral blood mononuclear cells (PBMC) were isolated using ficoll separation. CD56^+^CD3^-^ NK cells were isolated from frozen PBMCs using an untouched NK cell isolation kit (Miltenyi Biotec, Germany). 1 x 10^6^ NK cells were cocultured in a 24-well plate with 0.5 x 10^6^ irradiated K562 cells expressing membrane-bound interleukin-21 and 4-1BB-ligand (K562^F^) in culture medium as described previously (5). Culture medium consisted of IMDM (Lonza, Switzerland) supplemented with 5% heat-inactivated FBS (Gibco, ThermoFisher Scientific, USA), 5% human serum (Sanquin, the Netherlands), 1.5% L-Glutamine (Lonza), 1% penicillin/streptomycin (Lonza). When indicated, culture medium was supplemented with a cytokine mix, consisting of 5 ng/mL IL-15 (Miltenyi Biotec, Germany) and 100 IU/mL IL-2 (Novartis, Switzerland). On day 3 post stimulation, NK cells were transduced with retroviral vector encoding for different murinized TCRs (muTCR) combined with CD8αβ. Transduced cells were MACS (Milteny Biotec) enriched for CD8β on day 7, and immediately re-stimulated with irradiated K562^F^. After 2 days, enriched muTCR-CD8αβ positive NK cells were transduced with either a retroviral construct encoding for CD3ζγϵδ or CD3ζγϵδ combined with soluble IL-15. Transduced NK cells were enriched for muTCR expression on day 14 by MACS and subsequently re-stimulated. NK cells were expanded for 7 days and frozen down in liquid nitrogen. Functional assays were performed between day 7 and 14 after the last re-stimulation.

### Viral constructs and retrovirus production

**Figure 1:**
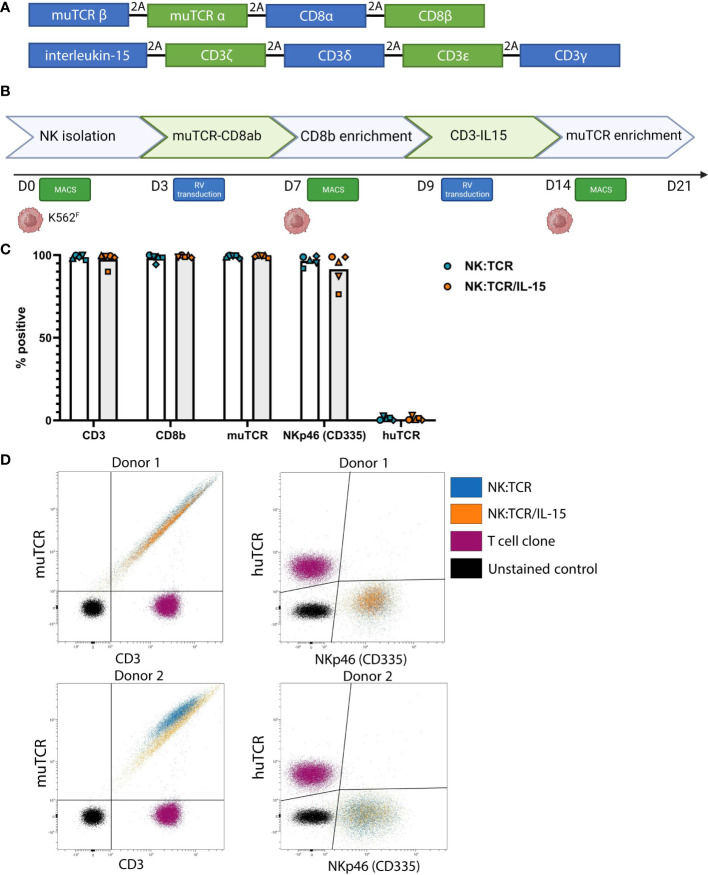
Generation of NK:TCR/IL-15 cells following a two-step retroviral production protocol. **(A)** Overview of vector sequence **(B)** Schematic of 21-day production protocol to generate TCR expressing NK cells that secrete soluble IL-15 (NK:TCR/IL-15). **(C)** Expression of introduced genes, CD3, CD8b, muTCR, NKp46, and huTCR. Each symbol represents one donor (N=5) **(D)** Expression of muTCR, CD3 and huTCR and NKp46 (CD335) of 2 different donors.

BOB1-B7 TCR (TRAV13 and TRBV4) and CMV-A2 TCR (TRAV18 and TRBV13) pLZRS retroviral vectors were constructed by linking the codon-optimized variable α and β chains to cysteine modified murine TCR α and β constant domains (muTCR) as described previously (18). Subsequently, the muTCRs were linked via 2A self-cleaving sites to CD8αβ (**Figure 1A**) (6, 19). Codon optimized CD3 invariant chains and soluble IL-15 were linked via 2A self-cleaving sites in the following order: IL-15, CD3ζ, CD3δ, CD3ϵ, CD3γ into the Moloney murine leukemia virus based pLZRS retroviral vector (**Figure 1A**, **Supplementary Table 1**) (20, 21). Subsequently, virus supernatant was made as previously described (5).

### Retroviral transduction

24-well flat-bottom non-tissue culture-treated plates (Greiner Bio-One, Austria) were coated with 30 ug/mL Retronectin (Takara, Kusatsu, Japan) and blocked with 2% human serum albumin (Sanquin, Amsterdam, The Netherlands). pLZRS retroviral supernatant was thawed and spun on Retronectin-coated wells at 3000g for 20 minutes at 4°C. Viral supernatant was then removed and 0.5 x 10^6^ NK cells were added to each well in 1 ml of NK-M. After 24 hours, transduced NK cells were transferred to tissue culture-treated culture plates and further expanded in NK-M. This method was previously described by Morton et al. (5, 19).

### Expansion of cryopreserved NK:TCR cells

Cryopreserved NK:TCR and NK:TCR/IL-15 cells were thawed and weekly stimulated with K562^F^ in culture media with or without a cytokine mix (IL-15 and IL-2). Expansion of the different NK:TCR cells was assessed by live-cell counting using Eosine every 7 days.

### Cytokine production

To assess cytokine production, 1 x 10^6^/ml NK:TCR and NK:TCR/IL15 were cocultured with or without K562^F^ cells in culture medium at an 1:1 Effector : Target (E:T) ratio in a 96-well round-bottom culture plate, in triplicate. After 24 hours supernatant was harvested. To assess the IL-15 concentration, the supernatants of the triplicates were pooled and measured with U-PLEX assay for human IL-15 (Meso Scale Diagnositics) following the manufacturer’s instructions and analyzed with corresponding software. In addition, IFNγ production by the different transduced NK cells was measured in the culture supernatant by IFNγ ELISA (Diaclone), according to manufacturer’s instructions.

### Target cell culture

Transformed EBV-LCLs (JY, HHC, IZA) and tumor cell lines, UM9 (multiple myeloma; MM), U266 (MM) and K562 were cultured in Iscove’s Modified Dulbecco’s Medium (IMDM) (Lonza, Switzerland) supplemented with 10% FBS (Gibco, Thermo Fisher Scientific, USA) 1.5% 200mM L-glutamine (Lonza, Switzerland), 1% 10,000U/ml penicillin/streptomycin (Lonza, Switzerland). The acute B-cell lymphoblastic leukemia (B-ALL) cell line, ALL BV, was obtained from patient material and cultured in IMDM (Lonza, Switzerland) with serum free supplement (22).

### Cytotoxicity assay

Cytotoxicity was assessed by flow cytometry-based cytotoxicity assay. Target cells were transduced with retroviral vector encoding for dTomato-Red, and dTomato-Red positive cells were subsequently sorted by fluorescence-activated cell sorting (FACS). A total of 30.000 dTomato-Red positive target cells were cocultured with 30.000 NK : TCR/IL15 or NK:TCR cells in round-bottom 96-well plates. After overnight coculture, cells were harvested, washed, and resuspended in 80μL FACS buffer containing Sytox blue live-dead marker (ThermoFisher Scientific). After a five minute incubation on ice, 40μL of sample (pre-set fixed volume criteria) was measured on a 3-laser Aurora at a consistent high flow rate of 65 µL/min (Cytek Biosciences). Flow cytometry data was unmixed using Spectroflo (Cytek Biosciences) and analyzed using FlowJo v10.7.1 (BD Biosciences). A live-dead gate was set on living cells and thereafter target cells were gated on dTomato-Red positive (see **Supplementary Figure 1** for a gating example). Percentage of cytotoxicity was calculated as the number of living targets cells after co-culture divided by living untreated targets cells, that were not co-incubated, using the following formula: $\left(1-\frac{number\;of\;living\;target\;cells\;after\;co-culture}{number\;of\;living\;untreated\;target\;cells}\right)*100$ . Each experiment was performed in duplo.

### NSG xenograft model

On day 0, male or female NOD.Cdg-*Prkdc*(scid)*Il2rg*(tm1Wjl/Szj (NOD *scid* gamma, NSG) mice (The Jackson Laboratory) were intravenously (i.v.) injected with 2 x 10^6^ luciferase-positive multiple myeloma U266 cells in PBS supplemented with 2% heat-inactivated FBS (Gibco, ThermoFisher Scientific, USA). After four days, to confirm localized tumor outgrowth in the bone marrow, tumor outgrowth of U266 cells was measured after subcutaneous (s.c.) injection of 150uL 7.5mM D-luciferine (Cayman Chemical) using a CCD camera (IVIS Spectrum, PerkinElmer). Subsequently, mice were treated with 5 x 10^6^ NK:TCR or NK:TCR/IL-15 cells, TCR specificity and number of mice are mentioned in corresponding figures. Tumor outgrowth was measured at regular intervals. Mice were sacrificed at day 40 or when mice reached an average luminescence of 1 x 10^7^ p/s/cm^2^/sr. Organs were collected and stained with immunohistochemistry when indicated. These studies were approved by the national Ethical Committee for Animal Research (AVD116002017891) and performed in accordance with Dutch laws for animal experiments. The number of mice included was based on a two-tailed, significance level of (α) 0,01, a power (π) of 80% and a standard deviation (σ) of 20%, with an expected effect size of 70%.

### Immunohistochemical analysis

Luciferase expression of U266 cells in FFPE tissue slides was analyzed with primary Goat anti-Human antibodies against Luciferase (AB181640, Abcam, Cambridge). A monoclonal Mouse anti-Human CD3 antibody (AB699, DAKO, Netherlands) was used to stain infiltration of NK:TCR/IL-15 cells. Secondary visualization was performed using a 3,3′-Diaminobenzidine(DAB) based standard laboratory procedure.

### Flow cytometry

FACS analysis was performed on either a 3 or 5-laser Aurora (Cytek Biosciences). For each stain, 50.000 NK cells were aliquoted into 96-well V-bottom plates, washed and incubated with 10uL antibody mix for 15 min at RT. An overview of antibodies used can be found in **Supplementary Table 2**. Flow cytometry data was unmixed using Spectroflo (Cytek Biosciences) and analysis was done using the OMIQ software from Dotmatics (www.omiq.ai, www.dotmatics.com).

### Statistics

Statistical analysis was performed using GraphPad Prism 7 software. Statistical tests used are mentioned in corresponding figures.

## Results

### Engineering of NK:TCR/IL-15 cells

NK cells were isolated from previously frozen PBMC of five different donors. Isolated NK cells were stimulated with irradiated K562^F^ cells, expressing membrane bound IL-21 and 4-1BB-ligand, and retrovirally transduced with different TCR-CD8 viral vectors, enriched and restimulated, and subsequently transduced with viral vectors encoding the different subunits of the CD3 complex and soluble IL-15 (**Figure 1A**). Following this two-step transduction protocol, as described previously by Morton et al, and depicted in **Figure 1B**, we genetically engineered highly pure populations of NK cells expressing muTCR, CD8 and CD3, accompanied by insertion of the IL-15 gene (NK:TCR/IL-15) (**Figure 1C**) (5). In these experiments, the constant region of each transgenic TCR was murinized (muTCR) to allow distinction between any contaminating T cells present in the culture, expressing the huTCR, which may influence functional data. This alteration does not affect any function of the TCR (18). Eventually, in the final products, less than 3% huTCR+ T cells were present. Despite the introduction of an additional gene, expression of the TCR/CD3 complex did not differ between NK:TCR and NK:TCR/IL-15 products (**Figure 1D**). Furthermore, no significant differences were observed in phenotype between NK:TCR and NK:TCR/IL-15 cells using a multispectral flow panel, including several activation, inhibition and adhesion markers (**Supplementary Figure 2**).

### NK:TCR/IL15 cells demonstrate robust proliferation in a cytokine-free environment

**Figure 2:**
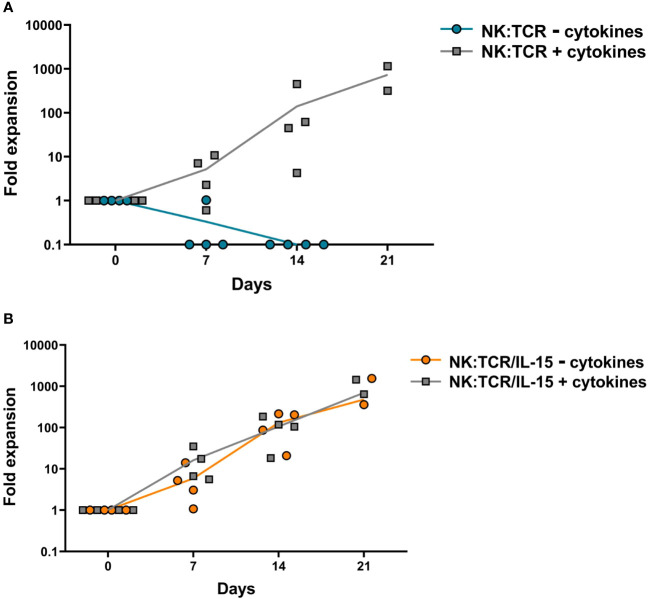
Expansion kinetics of NK:TCR/IL-15 cell products. Final products (N=4) (day 21 after isolation) were weekly stimulated with K562^F^ cells in culture media with or without suppletion of cytokines (IL-15 and IL-2). At each time point the cell number was determined by counting total viable cells present in the culture. **(A)** NK:TCR products **(B)** NK:TCR/IL-15 products. The indicated line represents the mean expansion per condition per product type.

To explore the expansion potential of NK:TCR/IL-15 cells, both NK:TCR and NK:TCR/IL15 were re-stimulated weekly with K562^F^ cells and cultured for at least 14 days in culture medium with or without suppletion of cytokines (IL-15 and IL-2). NK:TCR cells proliferated strongly upon stimulation with K562^F^ cells when cultured in medium supplemented with cytokines (**Figure 2A**), but were not able to proliferate when cultured in medium only, resulting in no viable NK cells present after 14 days of culture. In contrast, NK:TCR/IL-15 cells were able to proliferate in culture medium without cytokines and showed a similar expansion kinetic compared to NK:TCR cells cultured with cytokines. Additionally, NK:TCR/IL-15 proliferated at a similar rate whether cultured in presence or absence of cytokines in the culture medium (**Figure 2B**). This suggests that NK:TCR/IL-15 cells produce sufficient IL-15 to sustain their proliferation, without the need for suppletion of IL-15 or IL-2.

### Cytokine production by NK:TCR/IL-15 cells

**Figure 3:**
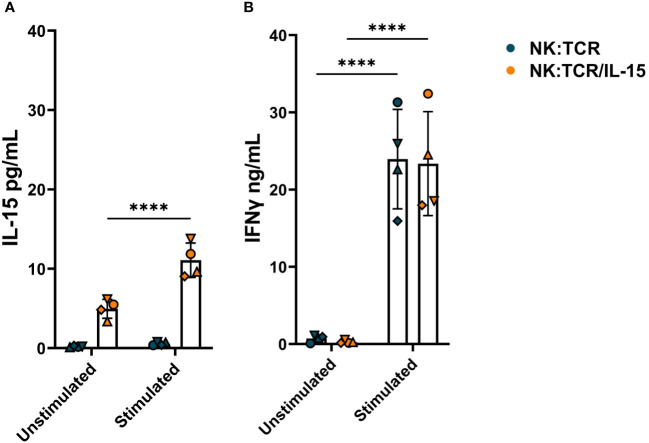
Cytokine production by NK:TCR/IL-15 cells. 1 x 10^6^/ml NK:TCR and NK:TCR/IL-15 cells were stimulated with K562^F^ cells in a 1:1 effector:target (E:T) ratio for 24 hours. **(A)** IL-15 production was measured by a mesoscale discovery assay and **(B)** IFNγ production was assessed by ELISA. All individual NK cell products derived from different donors and were matched during manufacturing and experiment (n=4). Each symbol represents a NK:TCR or NK:TCR/IL-15 product from a different donor (N=4). Error bars represent mean and SD of different donors. Statistical test used was two-way ANOVA with Sidak’s multiple comparisons *post hoc* test (**** = p<0.0001).

To determine the IL-15 secretion by NK:TCR/IL-15 cells, 1 x 10^6^/ml engineered NK cells were cultured alone or with irradiated K562^F^ cells at an E:T ratio of 1:1 in culture medium for 24 hours. As shown in **Figure 3A**, NK:TCR/IL-15 cells from four different donors demonstrated on average a baseline level of 5pg/mL secreted IL-15, whereas no IL-15 could be detected in the supernatant of NK:TCR cells. Stimulation with K562^F^ resulted in a doubling of IL-15 production by the NK:TCR/IL-15 cells, although the concentration of IL-15 in the supernatant did not exceed 15 pg/mL (**Figure 3A**). As a control for cytokine production IFNγ secretion was measured. After stimulation with K562^F^ cells, IFNγ production was strongly increased by both NK:TCR and NK:TCR/IL-15 cell products (**Figure 3B**).

### Incorporation of soluble IL-15 supports TCR-mediated cytotoxicity

**Figure 4:**
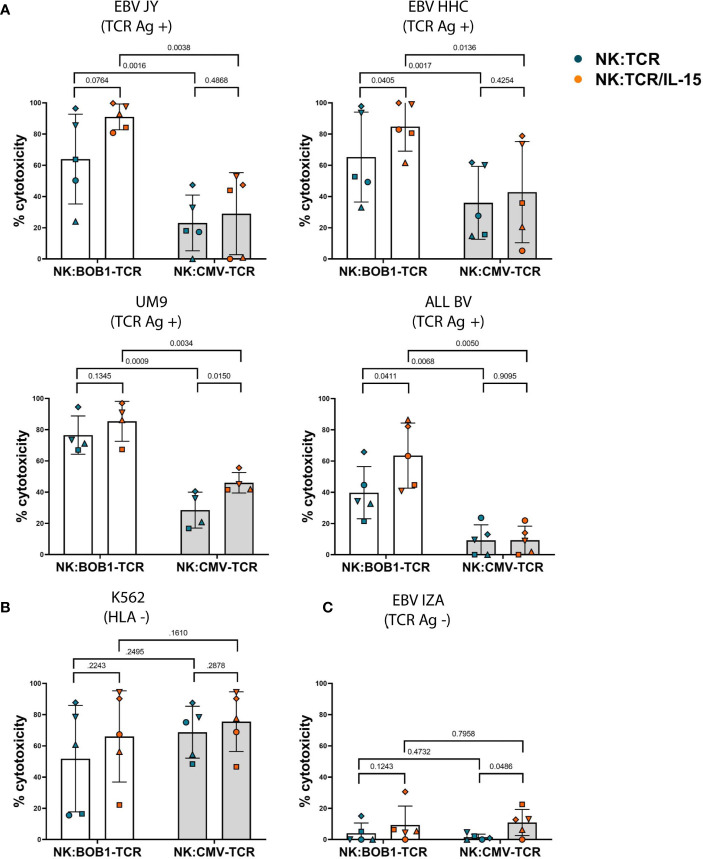
Increased TCR- and NK-mediated cytotoxicity by NK:TCR/IL-15 cells. NK:TCR and NK:TCR/IL-15 cells expressing either the HLA-B*07:02 restricted, BOB1-specific TCR or the HLA-A*02:01 restricted, CMV-specific control TCR were cocultured, at a 1:1 effector:target ratio, in culture medium with dTomato-positive transformed EBV-LCLs and tumor cell lines: **(A)** TCR Ag positive (HLA-B*07:02 +/BOB1+) targets: EBV JY, EBV HHC, UM9 (MM) and ALL BV (B-ALL). **(B)** HLA negative target K562 or **(C)** TCR Ag negative (HLA-B*07:02 -/BOB1+) target EBV IZA. All individual NK cell products derived from different donors and were matched during manufacturing and experiment (n=5). Each symbol represents a different donor (N=5). Statistical test used was a paired t-test, the p-value of each comparison is plotted.

To investigate whether autonomous production of IL-15 would influence the cytotoxic capability of NK:TCR cells, we transduced NK cells with an HLA-B*07:02 restricted TCR targeting a BOB1 derived peptide (herein referred to as BOB1-TCR). BOB1 is a B-cell lineage specific transcription factor uniformly expressed in malignant B cells, including multiple myeloma (MM), and demonstrated to be essential for the survival of MM (6, 23). As previously shown by Morton et al. NK:BOB1-TCR cells successfully target TCR Ag positive (BOB1+, HLA-B7+) tumor cells. To assess the beneficial effect of IL-15 production by engineered NK:TCR cells, NK:BOB1-TCR/IL-15 and NK:BOB1-TCR cell products generated from five different donors were cocultured overnight in culture medium with a panel of various TCR Ag positive (BOB1+, HLA-B7+) target cells: B-cell lines, EBV-JY and EBV-HHC, MM cell line UM9 and B-ALL cell line ALL BV (BOB1 and HLA-B7 expression; **Supplementary Figure 3**).NK:BOB1-TCR/IL-15 cells tested against this panel of TCR Ag positive (BOB1+, HLA-B7+) targets demonstrated to be as effective or even more effective compared to NK:BOB1-TCR cells (**Figure 4A**). As a control, both NK:BOB1-TCR products were cocultured with a TCR Ag negative (BOB1+, HLA-B7-) target, EBV-IZA and the HLA negative K562 cell line, known for its high sensitivity to NK cells. Both products demonstrated cytotoxic activity against the HLA negative K562 target cells (**Figure 4B**), whereas the TCR Ag negative (BOB1+, HLA-B7-) cell line EBV-IZA was not killed by both NK:BOB1-TCR cell products (**Figure 4C**). When HLA-B7 was introduced in EBV-IZA, NK:BOB1-TCR/IL-15 were able to kill this EBV-LCL (**Supplementary Figure 4**). It is known that supplement of IL-15 to NK cells increases NK functionality, therefore we engineered NK cells with an irrelevant TCR against a CMV-derived peptide presented in HLA-A*02:01 (referred to as CMV-TCR) to assess NK-mediated cytotoxicity (24). The effector functions of NK:CMV-TCR/IL-15 cells were evaluated in comparison to NK:CMV-TCR cells, showing increased NK-mediated cytotoxicity of NK:CMV-TCR/IL-15 against some CMV TCR Ag negative targets (**Figure 4**). However, the overall trend of enhanced cytotoxicity was more prominent when comparing NK:BOB1-TCR/IL-15 to NK:BOB1-TCR cells. Furthermore, the comparison of NK:BOB1-TCR/IL-15 cells and control NK:CMV-TCR/IL-15 cells clearly confirmed that NK:BOB1-TCR/IL-15 cells mediate TCR-specific cytotoxicity. as previously seen in NK:BOB1-TCR cells when compared to NK:CMV-TCR cells. (**Figure 4A**). These results demonstrate that autonomous production of IL-15 by TCR engineered NK cells mainly improves TCR-mediated cytotoxicity.

### IL-15 enhances the TCR-mediated function of TCR engineered NK cells *in vivo*

**Figure 5:**
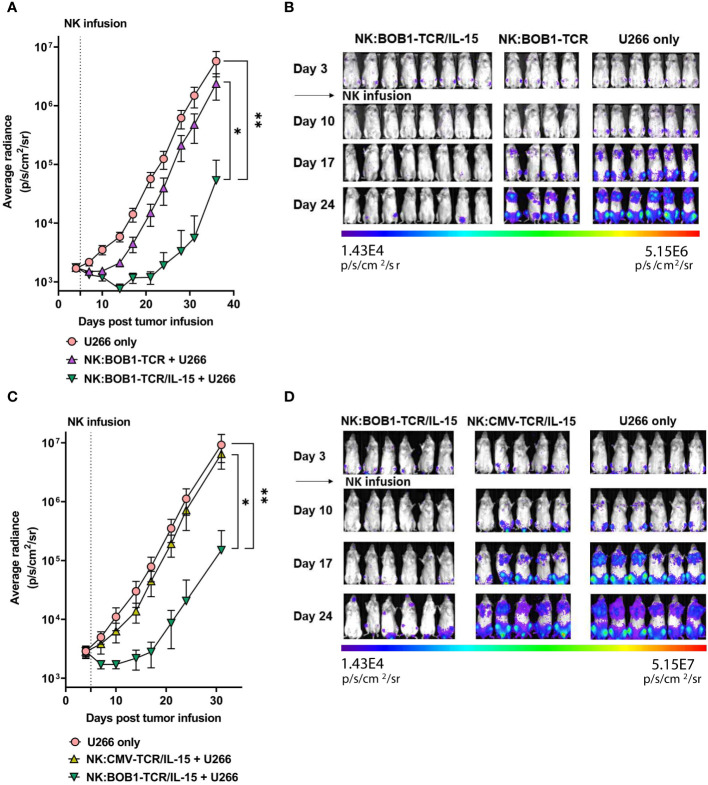
NK:TCR/IL-15 enhances tumor eradication in mice engrafted with multiple myeloma. NSG mice were infused intravenously with 2,0*10^6 HLA-B*07:02+ BOB1+ luciferase+ multiple myeloma U266 cells and after 4 days treated with **(A, B)** NK:BOB1-TCR/IL-15(n=8) or NK:BOB1-TCR (n=4) cells or with **(C, D)** NK:BOB1-TCR/IL-15(n=6) or control NK:CMV-TCR/IL-15(n=5) cells from the same donor. Non-treated mice (n=6) were taken along in every experiment (U266 only). For IVIS measurements the mice were injected s.c. with luciferin and measured for bioluminescence. Each error represent SD of the mean of all mice. Statistics depict mixed-effect analysis with Dunnett’s multiple comparisons *post hoc* test at endpoint (* = p<0.05, ** = p<0.01). BD) Bioluminescent images of mice at day 3, 10, 17 and 24.

To determine whether autonomous secretion of IL-15 enhances TCR-mediated function of NK cells *in vivo*, we compared the antitumor activity of NK:TCR and NK:TCR/IL-15 cells in an orthotopic MM model, as previously described (25). NSG mice were intravenously injected with the MM cell line U266 positive for the TCR Ag (BOB1+, HLA-B7+) resulting in bone marrow engraftment of the tumor. After four days the mice were treated with NK:BOB1-TCR or NK:BOB1-TCR/IL-15 cells, both NK cell products were generated from donor 2 (**Figure 1C**, dotted symbol **Figure 3** and **Figure 4**). During the first week after NK:TCR infusion both groups of mice treated with the genetically engineered NK cells (NK:BOB1-TCR and NK:BOB1-TCR/IL-15) demonstrated decrease in tumor burden, however only when mice were treated with NK:BOB1-TCR/IL-15 was the tumor burden significantly decreased in all mice (**Figures 5A, B**). Even, 5 out of 8 mouse remained free of tumor until 30 days post NK infusion, when the experiment was terminated (**Supplementary Figure 3**). To investigate whether this was due to increased NK- or TCR-mediated function of the engineered NK cells we performed an additional experiment in which the U266 engrafted NSG mice were treated after four days with NK:BOB1-TCR/IL-15 cells or NK:CMV-TCR/IL-15 cells as a negative control, again both NK products originated from donor 2. All mice treated with NK:BOB1-TCR/IL-15 showed a significant decrease in tumor burden directly after NK cell infusion compared with mice treated with NK:CMV-TCR/IL-15 and non-treated mice (**Figures 5C, D**). These results clearly demonstrate that autonomously produced IL-15 enhances the TCR-mediated function of TCR engineered NK cells *in vivo*.

### Accumulation of NK:BOB1-TCR/IL-15 cells *in vivo*

**Figure 6:**
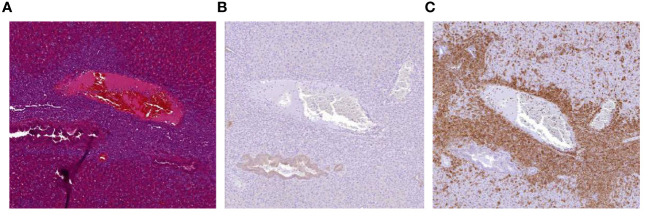
Accumulation of NK:BOB1-TCR/IL-15 cells after tumor clearance. Coupes from liver from a mouse first injected with 2*10^6 luciferase positive U266 tumor cells and treated with 5*10^6 NK:BOB1-TCR/IL-15 cells, 30 days post NK infusion. **(A)** HE staining. **(B)** luciferase DAB staining **(C)** human CD3 DAB staining.

Despite these promising observations we also encountered an unexpected and unwanted event within one of the *in vivo* experiments (depicted in **Figures 5A, B**). In this experiment, the beneficial antitumor activity of the NK:BOB1-TCR/IL-15 cells was accompanied by death of 3 out of 8 mice approximately 30 days after injection of NK:BOB1-TCR/IL-15 cells. Autopsy of the complete treatment group demonstrated accumulation of large clusters of cells in several organs, mainly lungs, spleen and liver in all eight NSG mice treated with NK:BOB1-TCR/IL-15 cells. Moreover, immunohistochemistry performed on these affected organs demonstrated that the clusters of cells were negative for luciferase, but positive for human CD3, confirming that these cells were NK:BOB1-TCR/IL-15 cells (**Figure 6**). In contrast, this phenomenon was not observed in mice treated with NK:BOB1-TCR cells that were generated from the same donor but did not secrete IL-15.To specify, these NK:BOB1-TCR/IL-15 and NK:BOB1-TCR cells were derived from the same NK cell isolation and TCR-CD8 transduction and were thereafter split into two groups. Subsequently, these NK cells were retrovirally transduced with either CD3 or CD3-IL15. The *in vitro* results of these specific donor-derived products are depicted in **Figure 1D** as donor 2 or depicted as a dotted symbol in all main figures. To investigate whether this phenomenon could reproducibly be observed and to gain more insight into the underlying mechanism, cryopreserved NK:BOB1-TCR/IL-15 cells from this specific experiment were expanded and injected into mice engrafted with U266. While antitumor activity was observed again, dysregulated growth of genetically engineered NK cells was not observed (**Supplementary Figure 5**). Eventually, within four independent experiments involving NK:BOB1-TCR/IL-15 cells, generated from the same donor (**Figures 6C, D**) as well as a different donor (data not shown), this unexpected and unwanted event was only observed once.

## Discussion

In this study we demonstrated that viral incorporation of a gene encoding for soluble interleukin-15 into engineered NK:TCR cells is feasible, whereby high expression of the TCR is maintained. As shown by Morton et al. equipping NK cells with a tumor specific TCR may override inhibition signals and boost therapeutic efficacy of adoptive NK cell transfer (5). By incorporating IL-15 into NK:TCR cells, engineered NK cells are able to proliferate *in vitro* without addition of extra cytokines. Furthermore, both the NK-mediated as well as TCR-mediated cytotoxicity of the NK:TCR cells is enhanced, resulting in improved persistence and performance of NK:TCR/IL-15 cells in an orthotopic multiple myeloma mouse model.

A number of studies have highlighted the beneficial effect of combining IL-15 stimulation with a CAR to engineer a more potent NK:CAR product (14, 15, 26–28). This study is the first to demonstrate successful engineering of NK:TCR cells with IL-15. Previously, Morton et al. showed that NK:BOB1-TCR cells successfully target TCR Ag positive (BOB1+, HLA-B7+) tumor cells (5). Our results reveal that the addition of IL-15 to the NK:BOB1-TCR product increased its cytotoxic profile against TCR Ag positive tumor cells, indicating improved TCR-mediated cytotoxicity. Furthermore, self-sufficient production of IL-15 by the NK:BOB1-TCR product showed a clear beneficial TCR-mediated effect *in vivo*, whereby NK:BOB1-TCR/IL-15 cells were able to clear the tumor and persevere this effect for a longer time compared to NK:BOB1-TCR cells. While prior studies mainly focused on the enhancement of CAR-mediated cytotoxicity by incorporating IL-15, we tried to dissect the additional benefit on both TCR-mediated as well as NK-mediated cytotoxicity. In our study, the known beneficial effect of IL-15 on NK-mediated cytotoxicity was confirmed by slightly improved effector functions of control NK:CMV-TCR/IL-15 cells, that exhibited an increased anti-tumor reactivity *in vitro* compared to NK:CMV-TCR cells. However, *in vivo*, this enhanced NK-mediated cytotoxicity was not enough for efficient tumor control. In contrast, NK:BOB1-TCR/IL-15 cells significantly controlled tumor outgrowth in this experiment. In addition, we also observed variability among donors within the *in vitro* cytotoxicity assays. To address this specific inter-donor variability inherent to NK cells, potentially influenced by differences in KIR-matching or expression of NKG2A and NKG2C, the same individual donor was used in the *in vivo* experiments (29). While our data revealed no differences in phenotype between NK:BOB1-TCR and NK:BOB1-TCR/IL-15 products derived from 5 donors (**Supplementary** **Figure 3**), we acknowledge that donor-specific characteristics of NK:TCR products could eventually amplify or diminish their therapeutic efficacy over time. Nevertheless, we can conclude that autonomous secretion of IL-15 by NK:TCR cells markedly improved TCR-mediated cytotoxicity *in vitro* and *in vivo* as well as enhancing NK-mediated cytotoxicity.

In addition to the prolonged anti-tumor reactivity by NK:BOB1-TCR/IL-15 *in vivo*, we observed in one out of four experiments an accumulation of NK:BOB1-TCR/IL-15 cells in several organs of treated mice, leading to death 30 days post NK infusion. Other studies also showed unexpected and preliminary death during one of their *in vivo* experiments. For instance, Lui et al. reported early deaths in 3 out of 5 mice following NK:CAR/IL-15 treatment, linked to complications related to release of inflammatory cytokines, hinting at a potential induction of CRS (15). However, in our experiment the NK:BOB1-TCR/IL-15 treated mice did not show any signs of toxicity in the early phase of the treatment, arguing against causes like CRS or neurotoxicity. Furthermore, Christodoulou et al. previously described preliminary death after treatment with NK:CAR/IL-15 cells, caused by severe inflammation due to dramatic NK-cell proliferation associated with high levels of IL-15 (14). A review by Ma et al. suggested that increased IL-15 production by NK:CAR/IL-15 cells could be a possible cause of this toxicity, since these NK:CAR/IL-15 cells reached an average baseline production of 200pg/mL IL-15 (30). In our experiments however, mice did not show any signs of severe inflammation, and NK:TCR/IL-15 cells did not reach a baseline IL-15 production level above 5pg/mL *in vitro*.

The absence of early inflammatory symptoms and low IL-15 levels argue against a form of CRS or IL-15 related inflammation in our experiment. In addition, accumulation of NK:BOB1-TCR/IL-15 was not observed in subsequent *in vivo* experiments. This combination of factors indicates that this was most likely an isolated event, possibly influenced by circumstantial factors such as viral integration or clonal expansion. Clonal expansion of CAR T cells has been described in several cases (31–33). In these studies, patients treated with CAR T cells showed a rapid response against the tumor, followed by the later accumulation of T cells. Subsequent TCR expression analysis revealed clonal expansion of specific clones, a phenomenon confirmed by gene insertion analysis, which identified disrupted genes involved in cell signaling. Unfortunately, confirming clonal expansion of our NK:BOB1-TCR/IL-15 cells is complicated due to two key factors. First, NK cells, in contrast to T cells, do not express a TCR or a TCR-like receptor with a unique sequence to identify individual clones. Secondly, gene insertion analysis of NK:TCR/IL-15 cells is complex due to the introduction of multiple genes encoded by two viral constructs containing similar LTR sequences, which are likely integrated multiple times. Consequently, we were unable to conduct a detailed analysis of the clonal origin of accumulated NK:BOB1-TCR/IL-15 cells. However, we speculate that multiple gene integration, possibly leading to gene disruption, in combination with autonomous cytokine production, even in low amounts, could be the cause of described unexplained events.

Therefore precautions remain essential for genetically engineered cellular products, particularly when factors promoting proliferation and growth are introduced. Future optimizations of our product may involve the introduction of an inducible caspase 9 safety switch, as was done by others before (3, 15, 26, 34, 35). Another consideration is the implementation of a TCR-induced localized secretion of IL-15, to ensure IL-15 production only occurs after antigen recognition (36). However, conditional secretion may not be sufficient to secure persistence when antigen load decreases, and it also does not prevent potential adverse events due to gene disruption.

To date, all available preclinical *in vivo* data are derived from NSG mouse models where human IL-15 is absent (14, 15, 26–28). In these models, all products benefit from IL-15, but toxicities have also been observed. Most NK:CAR products currently tested in the clinic are harnessed with IL-15 (NCT05110742, NCT05092451, NCT05922930). This offers great opportunities for the field of adoptive NK cell therapy, and these trials will provide valuable knowledge regarding safety issues of cytokine-secreting cellular therapeutics in a human setting.

In addition to data regarding safety issues, more insight into the performance of IL-15 secreting NK cells is needed. One study showed that incorporation of IL-15 into a NK:CAR product could overcome loss of metabolic fitness, a driver of tumor resistance (37). Unfortunately, this hypothesis was mainly based on preclinical data derived from NSG mice, since the cell numbers from clinical samples were too low to have enough power for thorough analysis. Nevertheless, the data supports the potential beneficial effect of IL-15 on NK cells in a non-nutritive environment. Additionally, understanding the effect of IL-15 secretion by tumor-specific NK cells in the tumor microenvironment is crucial. IL-15 is known to stimulate CD8 T cell proliferation and regulate tumor reactive lymphocyte numbers in the tumor microenvironment (11, 12, 38–40). Therefore, local secretion of IL-15 might shape the immune-modulating environment of the tumor and elicit T and NK-cell mediated anti-tumor responses.

Overall, precautions regarding safety remain since uncontrolled NK cell proliferation was observed in one *in vivo* experiment. This event highlights the importance of careful consideration and potentially more quality control for cytokine engineered lymphocytes before moving to clinical application. Nevertheless, optimization of adoptive NK therapy by autonomous production of IL-15 by the engineered NK cell might be an overall advantage. This paper showed that IL-15 engineered NK:TCR products enable proliferation of NK cells in an environment lacking cytokines, by secreting low levels of IL-15. In addition, primarily, autonomous IL-15 production improves NK:TCR/IL-15 functionality, whereby NK-mediated and TCR-mediated cytotoxicity is increased without causing off-target toxicity.

## Data availability statement

The raw data supporting the conclusions of this article will be made available by the authors, without undue reservation.

## Ethics statement

The studies involving humans were approved by the Institutional Review Board of the Leiden University Medical Center (approval number 3.4205/010/FB/jr) and the METC-LDD (approval number HEM 008/SH/sh). The studies were conducted in accordance with the local legislation and institutional, written informed consent was obtained from the patients/participants. The animal study was approved by the national Ethical Committee for Animal Research. The study was conducted in accordance with the local legislation and institutional requirements.

## Author contributions

EH: Formal analysis, Investigation, Methodology, Visualization, Writing – original draft. LM: Conceptualization, Funding acquisition, Methodology, Writing – review & editing. DR: Investigation, Writing – review & editing. AW: Investigation, Writing – review & editing. AV: Investigation, Writing – review & editing. JF: Supervision, Writing – review & editing. MH: Conceptualization, Funding acquisition, Methodology, Supervision, Visualization, Writing – review & editing.
