# Supplementary material for: Self-sufficient primary natural killer cells engineered to express T cell receptors and interleukin-15 exhibit improved effector function and persistence
Source: Front Immunol. 2024 Apr 16;15:1368290. doi: 10.3389/fimmu.2024.1368290 (PMC11058644; doi:10.3389/fimmu.2024.1368290)
Supplement: Supplementary file 1 [file DataSheet_1.docx]

Supplementary Material

**Self-sufficient primary natural killer cells engineered to express T cell receptors and interleukin-15 exhibit improved effector function and persistence Els P. van Hees^1^, Laura T. Morton^1^, Dennis F. G. Remst^1^, Anne K. Wouters^1^, Astrid Van den Eynde^2^, J. H. Frederik Falkenburg^1^ and Mirjam H.M. Heemskerk^1*^**

**^1^Department of Hematology, Leiden University Medical Centre (LUMC), Leiden, The Netherlands**

**^2^Center for Oncological Research (CORE), Integrated Personalized and Precision Oncology Network (IPPON), Wilrijk, Belgium**

*** Correspondence:**Mirjam H.M. Heemskerk
m.h.m.heemskerk@lumc.nl

# Supplementary Data

**Supplementary Table 1.** *Amino-acid sequences of vectors*

2A

TCR β-chain

TCR α-chain

CD8 α-chain

CD8 β-chain

Component of CD3

Interleukin-15

| muTCRαβ-CD8αβ (CMV) | MSIGLLCCAALSLLWAGPVNAGVTQTPKFQVLKTGQSMTLQCAQDMNHEYMSWYRQDPGMGLRLIHYSVGAGITDQGEVPNGYNVSRSTTEDFPLRLLSAAPSQTSVYFCASSSVTGTGNYGYTFGSGTRLTVVEDLRNVTPPKVSLFEPSKAEIANKQKATLVCLARGFFPDHVELSWWVNGKEVHSGVCTDPQAYKESNYSYCLSSRLRVSATFWHNPRNHFRCQVQFHGLSEEDKWPEGSPKPVTQNISAEAWGRADCGITSASYHQGVLSATILYEILLGKATLYAVLVSGLVLMAMVKKKNSGSGATNFSLLKQAGDVEENPGPMKSLRVLLVILWLQLSWVWSQILNVEQSPQSLHVQEGDSTNFTCSFPSSNFYALHWYRWETAKSPEALFVMTLNGDEKKKGRISATLNTKEGYSYLYIKGSQPEDSATYLCARNTGNQFYFGTGTSLTVIPDIQNPEPAVYQLKDPRSQDSTLCLFTDFDSQINVPKTMESGTFITDKCVLDMKAMDSKSNGAIAWSNQTSFTCQDIFKETNATYPSSDVPCDATLTEKSFETDMNLNFQNLSVMGLRILLLKVAGFNLLMTLRLWSSEGRGSLLTCGDVEENPGPATMALPVTALLLPLALLLHAARPSQFRVSPLDRTWNLGETVELKCQVLLSNPTSGCSWLFQPRGAAASPTFLLYLSQNKPKAAEGLDTQRFSGKRLGDTFVLTLSDFRRENEGYYFCSALSNSIMYFSHFVPVFLPAKPTTTPAPRPPTPAPTIASQPLSLRPEACRPAAGGAVHTRGLDFACDIYIWAPLAGTCGVLLLSLVITLYCNHRNRRRVCKCPRPVVKSGDKPSLSARYVEGRGSLLTCGDVEENPGPMRPRLWLLLAAQLTVLHGNSVLQQTPAYIKVQTNKMVMLSCEAKISLSNMRIYWLRQRQAPSSDSHHEFLALWDSAKGTIHGEEVEQEKIAVFRDASRFILNLTSVKPEDSGIYFCMIVGSPELTFGKGTQLSVVDFLPTTAQPTKKSTLKKRVCRLPRPETQKGPLCSPITLGLLVAGVLVLLVSLGVAIHLCCRRRRARLRFMKQFYK |
| --- | --- |
| muTCRαβ-CD8αβ (BOB1) | MGCRLLCCAVLCLLGAVPIDTEVTQTPKHLVMGMTNKKSLKCEQHMGHRAMYWYKQKAKKPPELMFVYSYEKLSINESVPSRFSPECPNSSLLNLHLHALQPEDSALYLCASSHGPASYEQYFGPGTRLTVTEDLRNVTPPKVSLFEPSKAEIANKQKATLVCLARGFFPDHVELSWWVNGKEVHSGVCTDPQAYKESNYSYCLSSRLRVSATFWHNPRNHFRCQVQFHGLSEEDKWPEGSPKPVTQNISAEAWGRADCGITSASYHQGVLSATILYEILLGKATLYAVLVSGLVLMAMVKKKNSGSGATNFSLLKQAGDVEENPGPMTSIRAVFIFLWLQLDLVNGENVEQHPSTLSVQEGDSAVIKCTYSDSASNYFPWYKQELGKGPQLIIDIRSNVGEKKDQRIAVTLNKTAKHFSLHITETQPEDSAVYFCAASKGSSNTGKLIFGQGTTLQVKPDIQNPEPAVYQLKDPRSQDSTLCLFTDFDSQINVPKTMESGTFITDKCVLDMKAMDSKSNGAIAWSNQTSFTCQDIFKETNATYPSSDVPCDATLTEKSFETDMNLNFQNLSVMGLRILLLKVAGFNLLMTLRLWSSGSGEGRGSLLTCGDVEENPGPATMALPVTALLLPLALLLHAARPSQFRVSPLDRTWNLGETVELKCQVLLSNPTSGCSWLFQPRGAAASPTFLLYLSQNKPKAAEGLDTQRFSGKRLGDTFVLTLSDFRRENEGYYFCSALSNSIMYFSHFVPVFLPAKPTTTPAPRPPTPAPTIASQPLSLRPEACRPAAGGAVHTRGLDFACDIYIWAPLAGTCGVLLLSLVITLYCNHRNRRRVCKCPRPVVKSGDKPSLSARYVEGRGSLLTCGDVEENPGPMRPRLWLLLAAQLTVLHGNSVLQQTPAYIKVQTNKMVMLSCEAKISLSNMRIYWLRQRQAPSSDSHHEFLALWDSAKGTIHGEEVEQEKIAVFRDASRFILNLTSVKPEDSGIYFCMIVGSPELTFGKGTQLSVVDFLPTTAQPTKKSTLKKRVCRLPRPETQKGPLCSPITLGLLVAGVLVLLVSLGVAIHLCCRRRRARLRFMKQFYK |
| CD3ζδεγ | MKWKALFTAAILQAQLPITEAQSFGLLDPKLCYLLDGILFIYGVILTALFLRVKFSRSADAPAYQQGQNQLYNELNLGRREEYDVLDKRRGRDPEMGGKPQRRKNPQEGLYNELQKDKMAEAYSEIGMKGERRRGKGHDGLYQGLSTATKDTYDALHMQALPPRGSGEGRGSLLTCGDVEENPGPMEHSTFLSGLVLATLLSQVSPFKIPIEELEDRVFVNCNTSITWVEGTVGTLLSDITRLDLGKRILDPRGIYRCNGTDIYKDKESTVQVHYRMCQSCVELDPATVAGIIVTDVIATLLLALGVFCFAGHETGRLSGAADTQALLRNDQVYQPLRDRDDAQYSHLGGNWARNKGSGATNFSLLKQAGDVEENPGPMQSGTHWRVLGLCLLSVGVWGQDGNEEMGGITQTPYKVSISGTTVILTCPQYPGSEILWQHNDKNIGGDEDDKNIGSDEDHLSLKEFSELEQSGYYVCYPRGSKPEDANFYLYLRARVCENCMEMDVMSVATIVIVDICITGGLLLLVYYWSKNRKAKAKPVTRGAGAGGRQRGQNKERPPPVPNPDYEPIRKGQRDLYSGLNQRRIGSGVKQTLNFDLLKLAGDVESNPGPMEQGKGLAVLILAIILLQGTLAQSIKGNHLVKVYDYQEDGSVLLTCDAEAKNITWFKDGKMIGFLTEDKKKWNLGSNAKDPRGMYQCKGSQNKSKPLQVYYRMCQNCIELNAATISGFLFAEIVSIFVLAVGVYFIAGQDGVRQSRASDKQTLLPNDQLYQPLKDREDDQYSHLQGNQLRRN |
| Interleukin15-CD3ζδεγ | MRISKPHLRSISIQCYLCLLLNSHFLTEAGIHVFILGCFSAGLPKTEANWVNVISDLKKIEDLIQSMHIDATLYTESDVHPSCKVTAMKCFLLELQVISLESGDASIHDTVENLIILANNSLSSNGNVTESGCKECEELEEKNIKEFLQSFVHIVQMFINTSGSGEGRGSLLTCGDVEENPGPPEFATMKWKALFTAAILQAQLPITEAQSFGLLDPKLCYLLDGILFIYGVILTALFLRVKFSRSADAPAYQQGQNQLYNELNLGRREEYDVLDKRRGRDPEMGGKPQRRKNPQEGLYNELQKDKMAEAYSEIGMKGERRRGKGHDGLYQGLSTATKDTYDALHMQALPPRGSGEGRGSLLTCGDVEENPGPMEHSTFLSGLVLATLLSQVSPFKIPIEELEDRVFVNCNTSITWVEGTVGTLLSDITRLDLGKRILDPRGIYRCNGTDIYKDKESTVQVHYRMCQSCVELDPATVAGIIVTDVIATLLLALGVFCFAGHETGRLSGAADTQALLRNDQVYQPLRDRDDAQYSHLGGNWARNKGSGATNFSLLKQAGDVEENPGPMQSGTHWRVLGLCLLSVGVWGQDGNEEMGGITQTPYKVSISGTTVILTCPQYPGSEILWQHNDKNIGGDEDDKNIGSDEDHLSLKEFSELEQSGYYVCYPRGSKPEDANFYLYLRARVCENCMEMDVMSVATIVIVDICITGGLLLLVYYWSKNRKAKAKPVTRGAGAGGRQRGQNKERPPPVPNPDYEPIRKGQRDLYSGLNQRRIGSGVKQTLNFDLLKLAGDVESNPGPMEQGKGLAVLILAIILLQGTLAQSIKGNHLVKVYDYQEDGSVLLTCDAEAKNITWFKDGKMIGFLTEDKKKWNLGSNAKDPRGMYQCKGSQNKSKPLQVYYRMCQNCIELNAATISGFLFAEIVSIFVLAVGVYFIAGQDGVRQSRASDKQTLLPNDQLYQPLKDREDDQYSHLQGNQLRRN |

**Supplementary Table 2.** *Overview of antibodies used*

| **CD designation** | **Alternative name** | **Fluorochrome** | **Type** | **Clone** | **Company** |
| --- | --- | --- | --- | --- | --- |
| CD137 | 41BB | APC | IgG1 | 4B4-1 | BD |
| CD158a | KIR2DL1 | FITC | IgM | HP-3E4 | BD |
| CD158b | KIR2DL2/3 | PE | IgG1 | GL183 | Beckman Coulter |
| CD158i | KIR2DS4 | BV786 | IgG2b | CH-L | BD |
| CD159a | NKG2A | BUV615 | IgG2a | 131411 | BD |
| CD159c | NKG2C | BV650 | IgG1 | 134591 | BD |
| CD16 | FcyRIII | FITC | IgG1 | NKP15 | BD |
| CD183 | CXCR3 | BUV496 | IgG1 | 1C6 | BD |
| CD184 | CXCR4 | BUV805 | IgG2a | 12G5 | BD |
| CD2 | CD2 | BV786 | IgG1 | RPA-2.10 | BD |
| CD27 | TNFRSF7 | AF700 | IgG1 | M-T271 | BD |
| CD279 | PD-1 | PE-Cy7 | IgG1 | EH12.1 | BD |
| CD28 | CD28 | APC-H7 | IgG1 | CD28.2 | BD |
| CD3 | CD3 | APC-H7 | IgG1 | SK7 | BD |
| CD314 | NKG2D | BV711 | IgG1 | 1D11 | BD |
| CD335 | NKp46 | BV421 | IgG1 | 9E2 | BD |
| CD38 | ADPRC1 | BV605 | IgG1 | HIT2 | Invitrogen |
| CD56 | NCAM1 | BV510 | IgG2b | NCAM16.2 | BD |
| CD62L | L-selectin | BUV395 | IgG2a | SK11 | BD |
| CD8β | CD8β | PE | IgG2a | 2ST8.5H7 | Beckman Coulter |
| CD94 | KLRD1 | PE | IgG1 | HP-3D9 | BD |
|  | huTCR | PerCP-Cy5.5 | IgG1 | IP26 | Biolegend |
|  | muTCR | APC | IgG2 | H57-597 | BD |

**Supplementary Figure 1.** *Gating strategy FACS-based killing assay.*

Gating of living ALL BV cells after O/N culture with or without co-incubation with effectors cells. Numbers (#) in the last plot indicate the counts of total living ALL BV cells, gated on FSC/SSC, Sytox Blue – and dTomato +.

**Supplementary Figure 2.** *Phenotype of NK:TCR/IL-15 cells*

NK:TCR and NK:TCR-sIL15 cells (day 10 post stimulation) were stained for different surface markers. Each symbol represent an NK:TCR(-sIL15) cell product from a different donor. Statistical test used was a multiple paired t-test, all comparisons were non-significant.

**Supplementary Figure 3.** *Relative expression of BOB-1, HLA class I and HLA-B*07 in target cells*

A) Relative mRNA expression of BOB-1 to housekeeping genes of target cells measured by qPCR B) Relative mean fluorescence intensity (MFI) of HLA-B*07 expression to the MFI of HLA class I expression of different target cells C) Cell surface expression of HLA class I and HLA-B*07, in green the HLA-B*07+ targets and in red the HLA-B*07- target EBV IZA. HLA negative cell line K562 (black) was used as a control.

**Supplementary Figure 4.** *NK:BOB1-TCR/IL-15 cells mediate cytotoxicity against EBV IZA cells transduced with HLA-B*07:02*

NK:BOB1-TCR/IL-15 cells expressing the HLA-B*07:02 restricted, BOB1-specific TCR was cocultured, at a 1:1 effector:target ratio, in culture medium with dTomato-positive EBV IZA (HLA-B*07:02 -/BOB1+) or EBV IZA transduced with HLA-B*07:02 (HLA-B*07:02 +/BOB1+). Each error represent SD of technical replicates (n=1).

**Supplementary Figure 5.** *NK:TCR/IL-15 enhances tumor eradication in mice engrafted with multiple myeloma*

NSG mice were infused intravenously with 2,0*10^6 HLA-B*07:02+ BOB1+ luciferase+ multiple myeloma U266 cells and after 4 days treated with NK:BOB1-TCR/IL-15(n=8) or NK:BOB1-TCR (n=4) cells or non-treated mice (n=6) (U266 only). For IVIS measurements the mice were injected s.c. with luciferin and measured for bioluminescence. The figure shows all individual mice depicted in the data of figure 6A.

**Supplementary Figure 6.** *NK:TCR/IL-15 enhances tumor eradication in mice engrafted with multiple myeloma*

NSG mice were infused intravenously with 2.0*10^6 HLA-B*07:02+, luciferase positive U266 multiple myeloma cells and after 4 days treated with NK:BOB1-TCR/IL-15 (N=3) deriving from the same production batch causing unexpected NK proliferation in vivo in a previous experiment (shown in figure 6AB and 7) or non-treated (U266 only). For measurement mice were injected s.c. with luciferin and measured for bioluminescence. Each error represent SD of the mean of all mice. Statistics depict mixed-effect analysis with Sidak’s multiple comparisons post hoc test at the endpoint.
