# Supplementary figures and images for: Self-sufficient primary natural killer cells engineered to express T cell receptors and interleukin-15 exhibit improved effector function and persistence
Source: Front Immunol. 2024 Apr 16;15:1368290. doi: 10.3389/fimmu.2024.1368290 (PMC11058644; doi:10.3389/fimmu.2024.1368290)

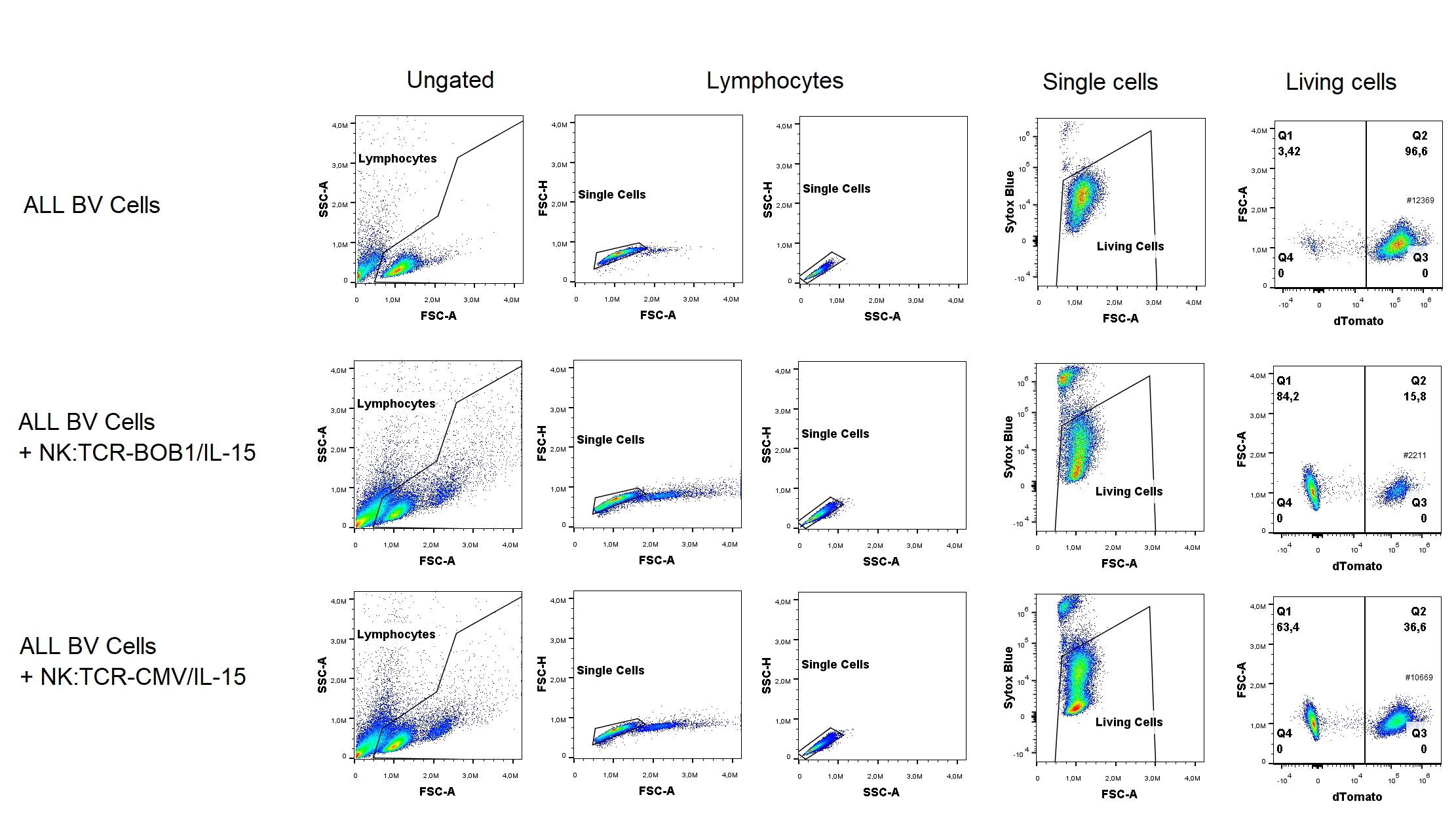

Supplement: Supplementary file 2 [file Image_1.tiff]

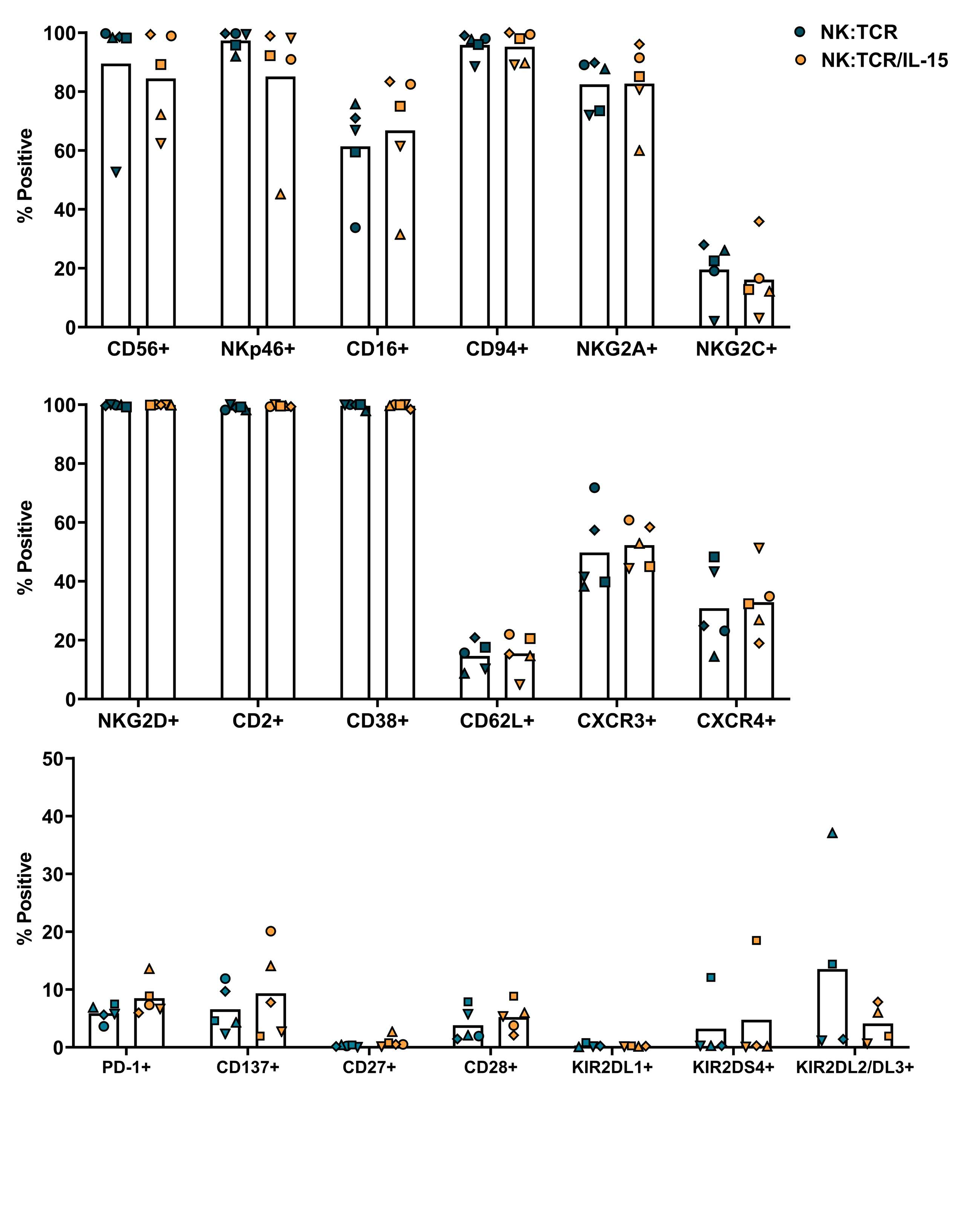

Supplement: Supplementary file 3 [file Image_2.tif]

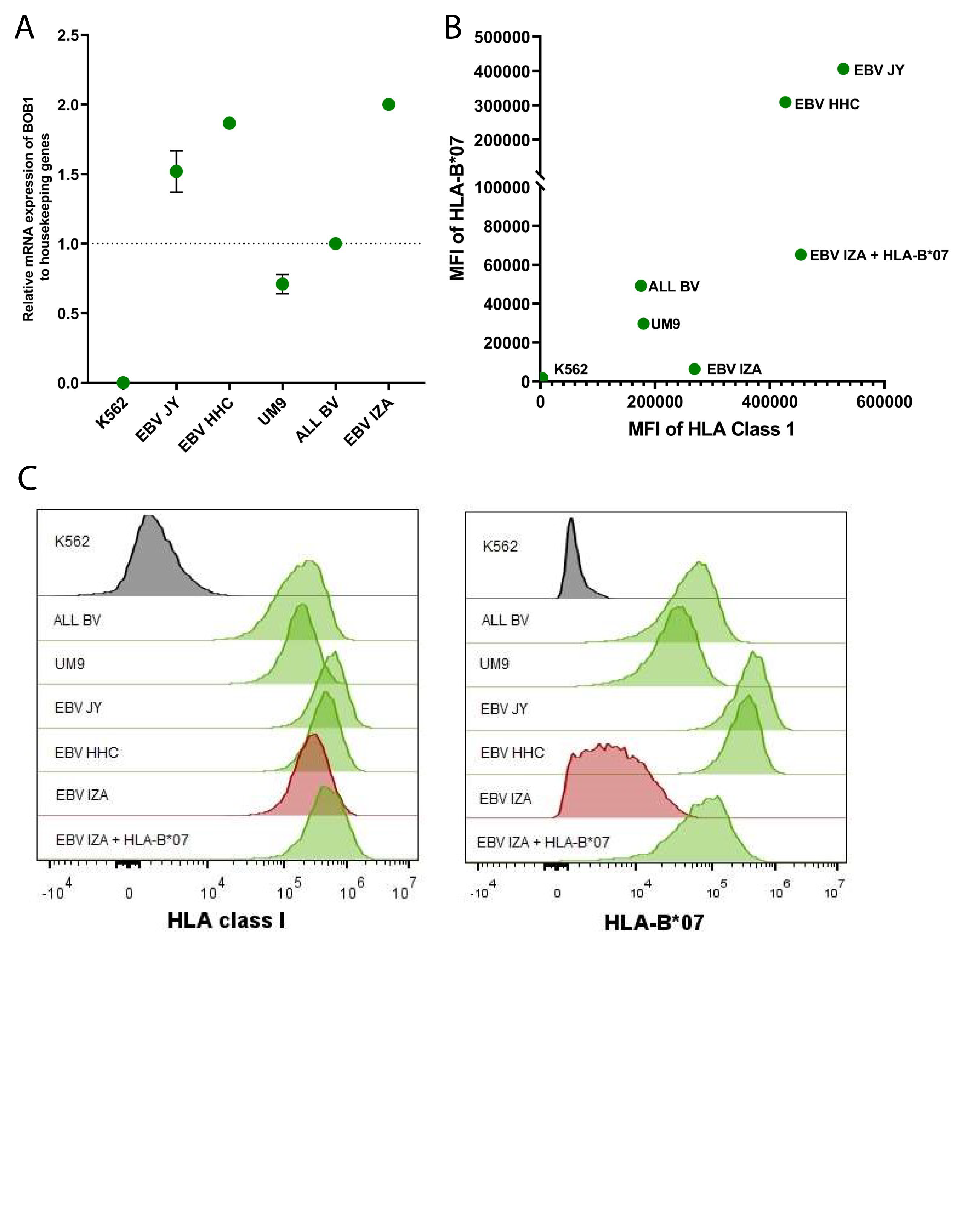

Supplement: Supplementary file 4 [file Image_3.tif]

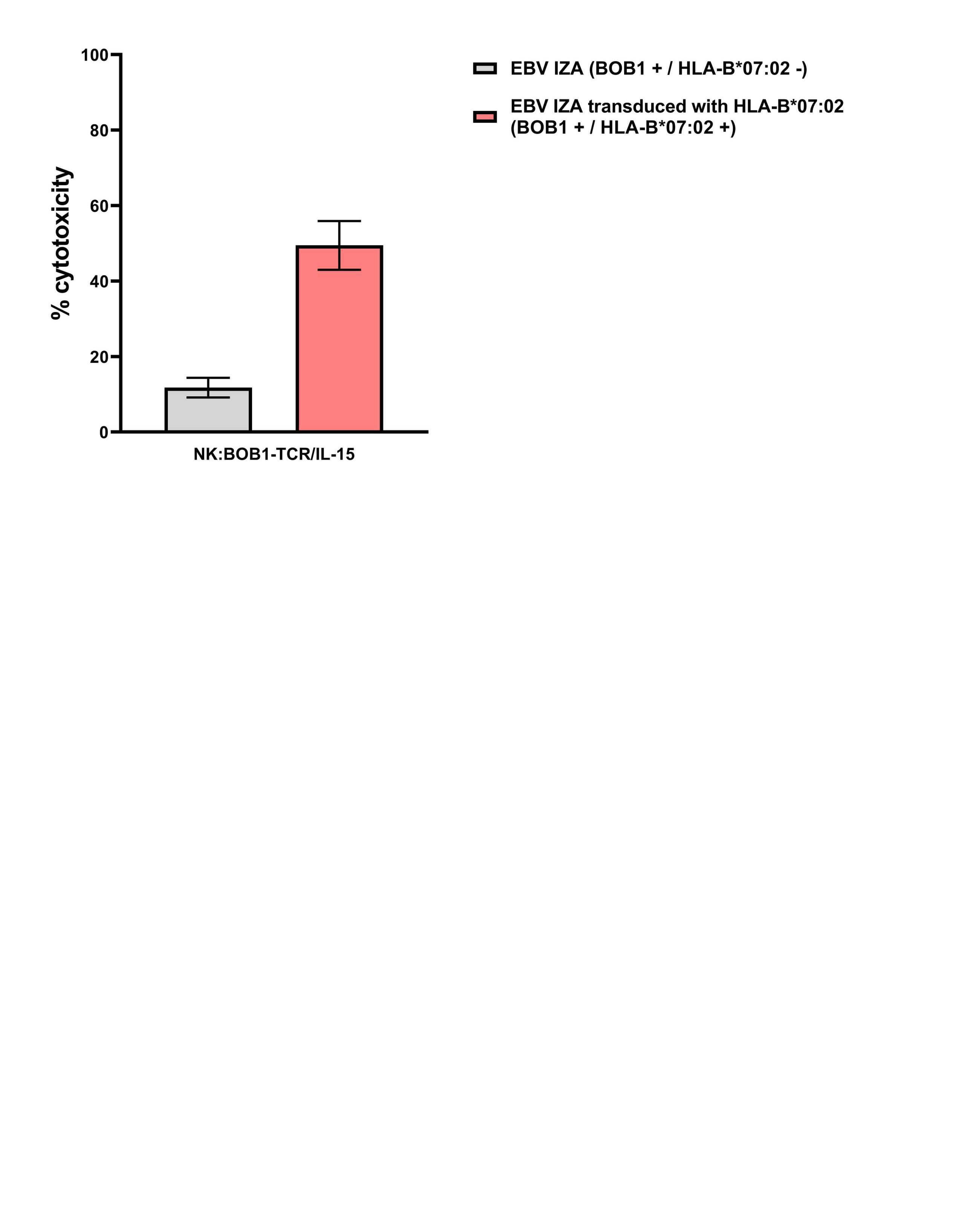

Supplement: Supplementary file 5 [file Image_4.tif]

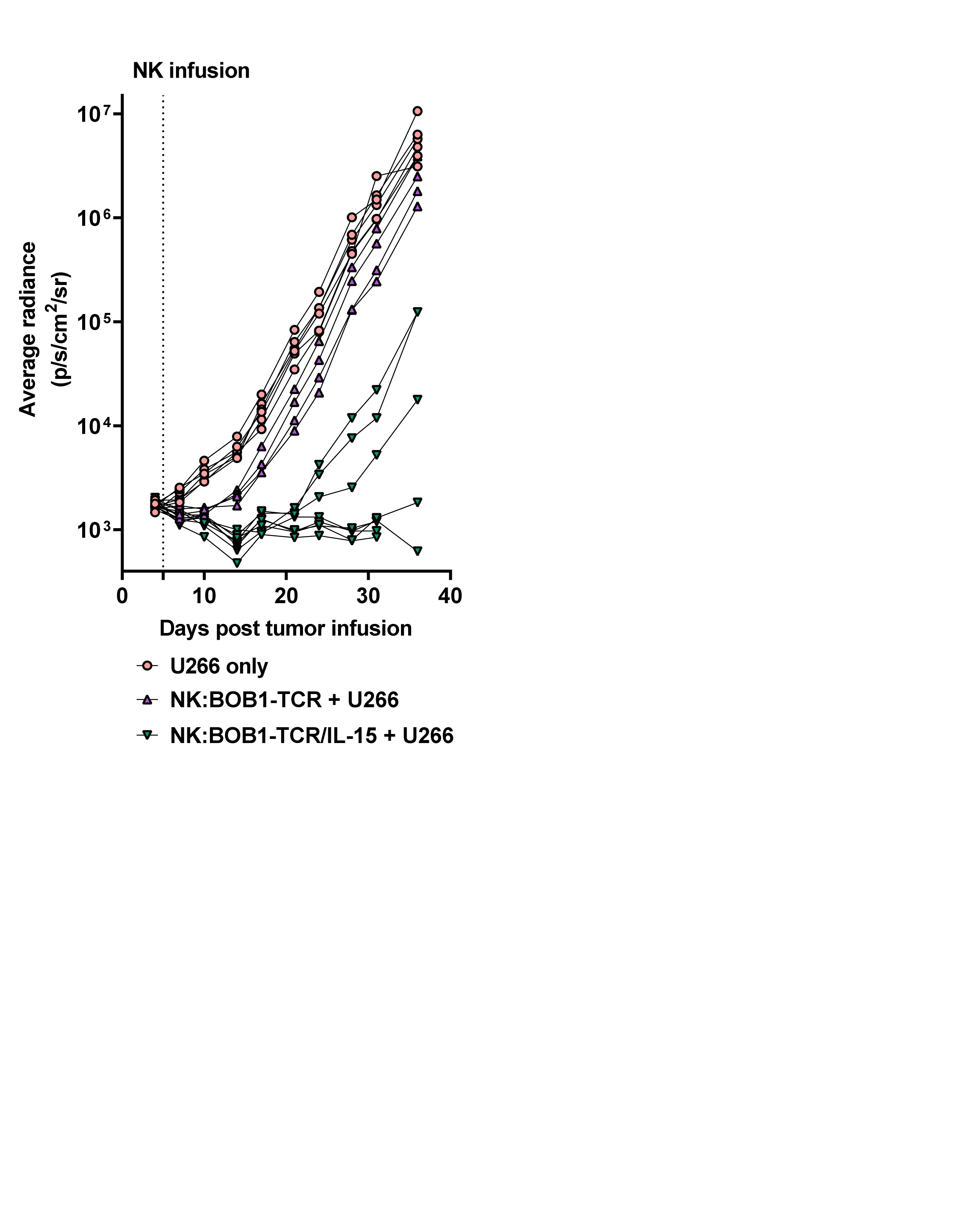

Supplement: Supplementary file 6 [file Image_5.tif]

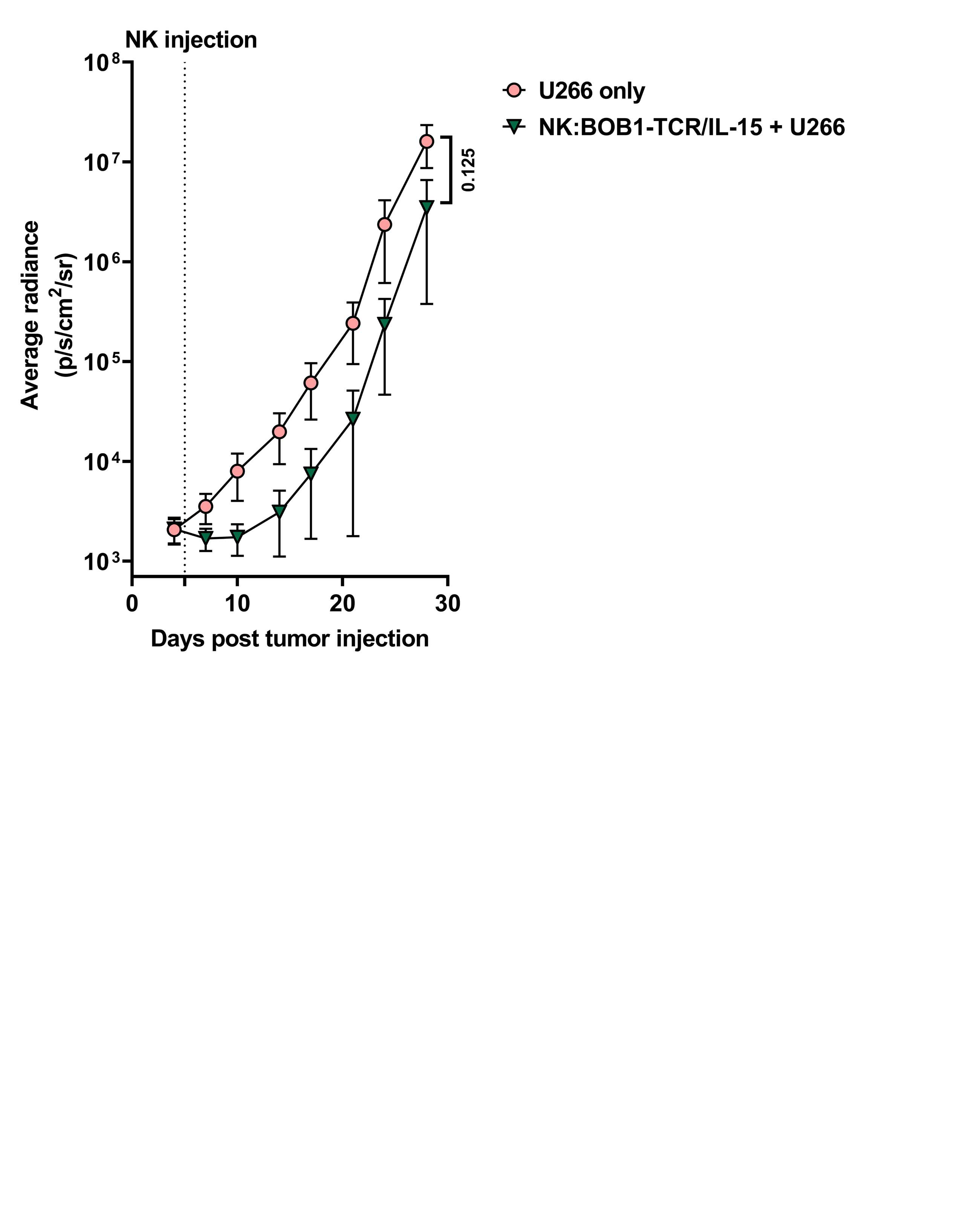

Supplement: Supplementary file 7 [file Image_6.tif]
